# Effects of Food and Temperature on *Drosophila melanogaster* Reproductive Dormancy as Revealed by Quantification of a GFP-Tagged Yolk Protein in the Ovary

**DOI:** 10.3389/fphys.2021.803144

**Published:** 2022-01-03

**Authors:** Yusuke Hara, Daisuke Yamamoto

**Affiliations:** Neuro-ICT Laboratory, Advanced ICT Research, National Institute of Information and Communications Technology, Kobe, Japan

**Keywords:** diapause, fruit fly, yolk protein, ovarian arrest, diet composition, GFP marker

## Abstract

When exposed to harsh environmental conditions, such as food scarcity and/or low temperature, *Drosophila melanogaster* females enter reproductive dormancy, a metabolic state that enhances stress resistance for survival at the expense of reproduction. Although the absence of egg chambers carrying yolk from the ovary has been used to define reproductive dormancy in this species, this definition is susceptible to false judgements of dormancy events: e.g. a trace amount of yolk could escape visual detection; a fly is judged to be in the non-dormancy state if it has a single yolk-containing egg chamber even when other egg chambers are devoid of yolk. In this study, we propose an alternative method for describing the maturation state of oocytes, in which the amount of yolk in the entire ovary is quantified by the fluorescence intensity derived from GFP, which is expressed as a fusion with the major yolk protein Yp1. We show that yolk deposition increases with temperature with a sigmoidal function, and the quality of food substantially alters the maximum accumulation of yolk attainable at a given temperature. The *Yp1::GFP* reporter will serve as a reliable tool for quantifying the amount of yolk and provides a new means for defining the dormancy state in *D. melanogaster*.

## Introduction

Many insects inhabiting the temperate zone overwinter by minimising metabolism and enhancing stress tolerance for survival under harsh environmental conditions. This specialised state enabling an organism to surpass unfavourable climate is known as diapause (Ragland et al., 2019; Saunders, 2020). Insects enter diapause well before the onset of extreme cold weather, often in response to anticipatory external cues, such as a short photoperiod and lower temperature; however, some species undergo obligatory diapause irrespective of environmental conditions (Denlinger, 2002; Beer and Helfrich-Förster, 2020; Saunders, 2020). Typically, diapausing individuals are immobile; however, the diapausing individuals of some species remain active yet reproductive physiological and behavioural traits are suspended, which is thus called reproductive diapause (Denlinger, 2002).

Among insects that have been the focus of biological research, *Drosophila melanogaster* has made unparalleled contributions to general biology due to its genetic tractability. Nonetheless, studies of diapause have been only marginally benefitted from *Drosophila* genetics, primarily because the shallow reproductive diapause of female *D. melanogaster* is difficult to detect, and substantial variations in the level of reproductive arrest across oocytes in a diapausing female complicate quantitative analysis. Despite this, several diapause studies in *Drosophila* showed that some of the natural populations in higher latitudes exhibit a greater tendency to undergo reproductive arrest under low temperature and/or a short photoperiod; alleles in the genes *PI3 kinase* (Williams et al., 2006), *timeless* (Tauber et al., 2007) and *couch potato* (*cpo*; Schmidt et al., 2008, see also Lee et al., 2011) were implicated in these phenotypic variations.

Reproductive diapause in females of a laboratory strain of *D. melanogaster* was first reported by Saunders et al. (1989). In their paper, reproductive diapause in this species was defined by the complete disappearance of yolk from the entire ovaries in a mature female fly after exposure to diapause-inducing conditions. Saunders et al. (1989) kept flies under the condition of a 10/14 l/D cycle at 12°C to induce diapause. Most recent works adopted the same criterion to define diapause in *D. melanogaster*, in which the absence or presence of yolk was judged by a visual inspection of unstained ovaries under a microscope, and the proportion of flies carrying solely previtellogenic oocytes was used to express the prevalence of diapause in a given fly population (Kubrak et al., 2014; Anduaga et al., 2018). Estimation of the diapause rate may suffer from at least two procedural limitations. First, the extent of ovarian arrest is only describable at the population level and quantitative differences in the maturity of oocytes among flies are not considered. Each fly is labelled as either diapausing or non-diapausing in an all-or-nothing manner, even though the amount of accumulated yolk varies from fly to fly in a graded manner. Second, a small amount of yolk in an oocyte can be overlooked in an unstained preparation, which will potentially lead to an overestimation of the diapausing rate.

Lirakis et al. (2018) added another caveat, which is that oocytes in flies are judged to be diapausing based on the conventional criterion of lacking yolk, not due to simple developmental arrest but because of the combination of arrest at the previtellogenic stage (stage 7 according to King, 1970) and stress-induced degeneration of early vitellogenic oocytes (stages 8 and 9), once produced. These authors noted that, although ovaries gain yolk when flies are kept under diapausing conditions for 2–4 weeks, oogenesis does not proceed beyond stage 9. Taking account of the fact that the ovary itself (more specifically follicle cells) produces a small amount of yolk by stage 9; then, starting at stage 10, fat bodies take over the role of follicle cells and synthesise a large amount of yolk, Lirakis et al. (2018) suggested that arrest just before stage 10, rather than arrest at stage 7 as established by the conventional criterion, should be adopted to define the dormancy state.

These considerations indicate that precise quantification of the yolk amount that accumulates in oocytes, particularly during stages 7–9 of previtellogenic and early vitellogenic oocytes, is pivotal for accurate estimation of the diapausing rate. This study was undertaken to develop an alternative means for defining reproductive dormancy in *D. melanogaster* females that is invulnerable to the aforementioned problems that are inherent when using the conventional method. We demonstrated that yolk protein 1 (Yp1) as a major constituent of yolk serves as a useful marker of the amount of yolk in an oocyte and in a whole ovary, which is detected by a GFP-tag attached to Yp1 and quantified by the GFP fluorescence intensity. With the aid of *Yp1::GFP*, we demonstrated that the feeding state and temperature, which affect dormancy in *D. melanogaster* (Ojima et al., 2018), strongly impacted yolk accumulation in the ovary, yet the stimulus–response dynamics revealed a range of varieties of stress responsiveness among individuals. The *Yp1::GFP*-aided estimation of the yolk amount in the ovary will provide reliable measures of ovarian arrest, even during shallow reproductive diapause.

## Materials and Methods

### Fly Strains and Cultures

*w^1118^* and *Yp1::GFP* (v318746, VDRC) were used in this study. The *Yp1::GFP* fly line used in this study is one of the fly-TransgeneOme lines, a fly resource that allows labelling of ~1,000 functional proteins with a C-terminal superfolder-GFP tag and what has been verified to recapitulate subcellular localization of the endogenous protein in the correct cell types at correct timing (Sarov et al., 2016). If not stated otherwise, flies were reared on a normal cornmeal–yeast medium with the following composition: 40 g cornmeal, 80 g dry yeast, 100 g glucose and 6 g agar dissolved in 1 litre of water supplemented with 5 ml propionic acid and 5 ml of 10% para-hydroxybenzoate. In the analysis of the effect of feeding status on the ovarian maturation, newly emerged virgin females were collected within 6 h of eclosion and were divided into two groups: flies in one group were immediately sacrificed for the examination of ovaries; the rest was reared on either a normal cornmeal–yeast medium or a nutrient-deficient medium prepared with 6 g agar and 1 L of water at 11°C. The recipes of yeast and plant foods were described in Brankatschk et al. (2018): The yeast food consisted of 20 g yeast extract, 20 g peptone, 30 g sucrose, 20 g glucose, 80 g dry yeast, 10 g agar, 6.3 ml propionic acid and 15 ml of 10% methylparaben per 1 L of water; the plant food consisted of 30 g beet sugar, 45 g malt, 2 g sunflower oil, 20 g peptone, 55 g cornmeal, 75 g glucose, 10 g agar, 6.3 ml propionic acid and 15 ml of 10% methylparaben per 1 L of water.

### Tissue Preparation

The ovaries were dissected in phosphate-buffered saline (PBS), fixed in 4% paraformaldehyde in PBS for 20 min at room temperature and washed three times in PBS with 0.5% Triton X-100 (PBT) for 30 min. Then, the tissues were mounted in 80% VECTASHIELD with 4′,6-diamidino-2-phenylindole (Vector Laboratories Inc.).

### Image Acquisition and Measurement of the GFP Signal Intensity

There were several advantages of using *Yp1::GFP* as a yolk reporter. First, subtracting the fluorescence signal intensity in the background from that throughout the entire ovary made it possible to unequivocally determine the amount of yolk in the ovary. Second, the fluorescence signal intensity reflected the absolute amount of yolk, which enabled the level of ovarian maturation of each fly to be determined on an absolute scale. Third, because of the above two, the average and variance of the ovarian maturity level of a fly population were determined and compared among different populations.

Confocal images were obtained with a LSM980 confocal microscope (Carl Zeiss). To obtain measurements of the GFP signal intensity, we acquired images using a M205FA automated stereomicroscope. Images in 1920 × 1440 pixel resolution were obtained using a DFC7000T colour CCD camera (Leica Microsystems) controlled by LasX software (Leica Microsystems). A metal halide bulb was used as an excitation light source, and GFP signals were detected by a GFP filter. The dark field and GFP images were captured for every sample in the exact same setting and were exported from LasX as TIFF files. The files were imported into ImageJ (National Institutes of Health). Fluorescence measurements were conducted for the region of interest (ROI), which was usually the entire ovary in this study. We manually defined the ROI with the wand tool in dark filed images and measured the GFP intensity in the green channel of GFP images in the following three areas: ‘Area a’ covered whole ovaries, whereas ‘Area b’ and ‘Area c’ represent control areas. To define Area b and Area c, we drew two circles with a diameter of 68.9 μm (30 pixels) at the germaria of the left and right ovaries. Germaria contain germ stem cells and proliferating daughter cells that harbour no yolk and thus serve as ‘blank’ control regions. The GFP signals derived from *Yp1::GFP* were obtained by subtracting the background fluorescence measured in the ‘blank’ control region from the total fluorescence outputs from the ROI. The subtracted value is normalised for the sizes of measured areas (Size_a_/Size_b_) and, when necessary, also for the body size differences (described by the factor *L*), yielding Intensity_GFP_. The factor *L* is the ratio of the anteroposterior body length of a test fly over the mean body length of all control flies in a given experiment. In the present study, scaling by the factor *L* was not applied.

### Statistical Analysis

Statistical analysis was carried out using Prism9 (GraphPad). Statistical significance among dormancy rates for different fly groups was evaluated using Fisher’s exact test. Statistical significance of the GFP intensity values among different fly groups was evaluated by the Kruskal–Wallis test with a post-hoc test. Statistical parameters are reported in the respective figure legends.

## Results

**Figure 1:**
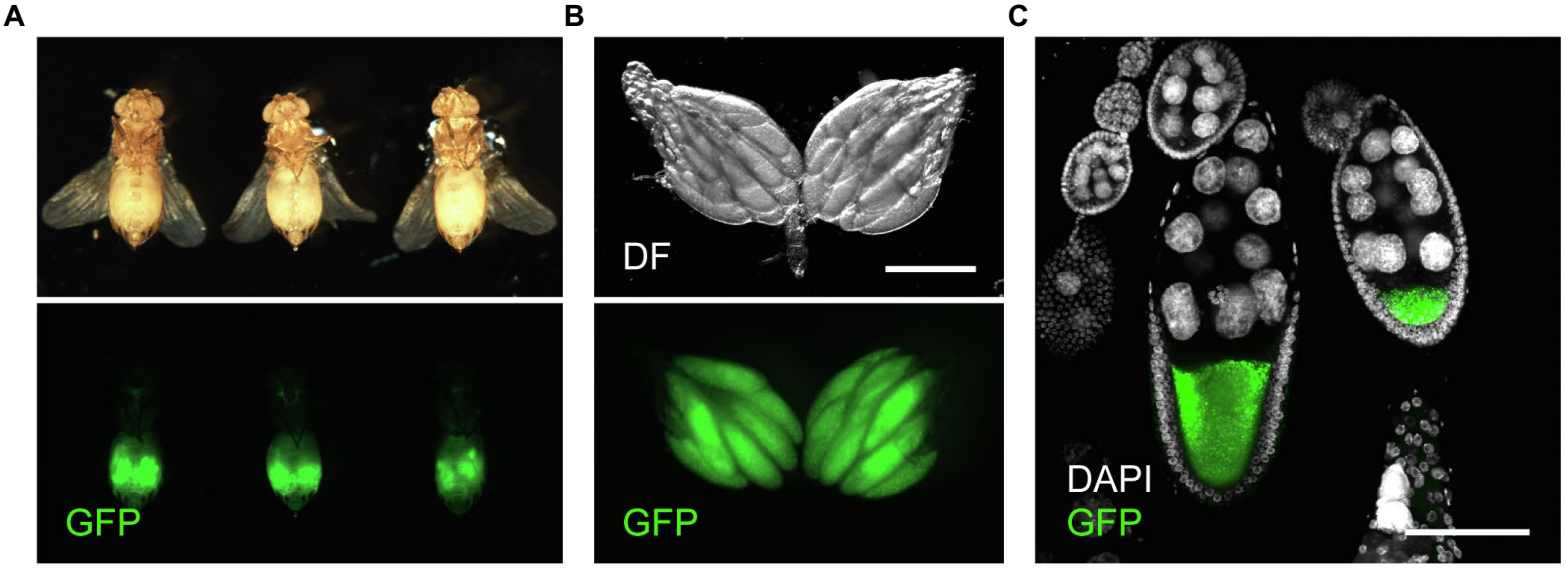
GFP expression in *Yp1::GFP* female flies. **(A)** Ventral views of three representative *Yp1::GFP* female flies (upper panels) and respective ovaries (lower panels) observed under a fluorescent microscope. **(B)** Representative images showing GFP fluorescence in the ovary. Scale bar: 500 μm. **(C)** Variabilities in the yolk accumulation levels in oocytes across ovarioles within a single ovary. Scale bar: 100 μm.

**Figure 2:**
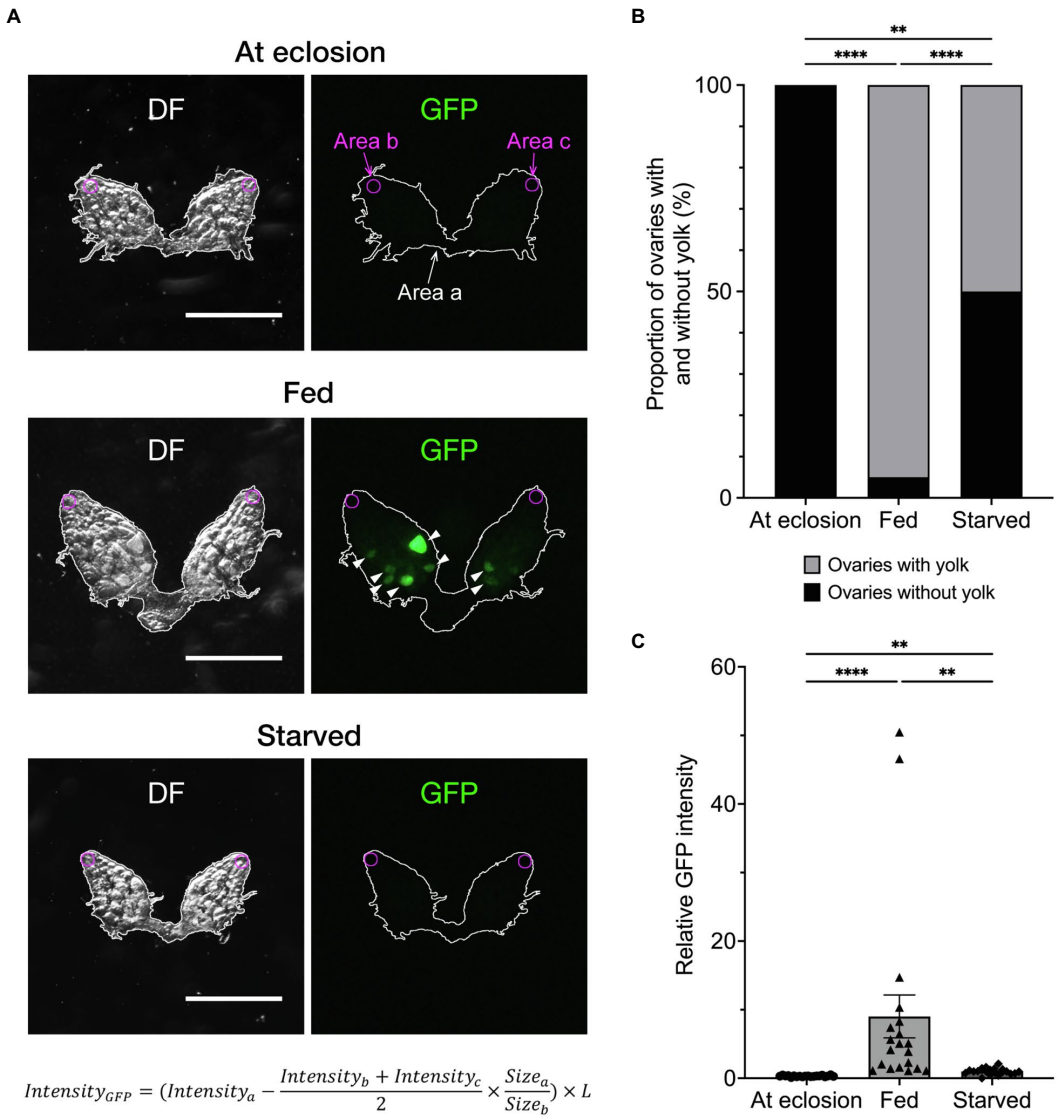
Effects of feeding on yolk accumulation in the ovary. **(A)** Representative images showing GFP fluorescence in the ovaries of an unfed fly at emergence (top), 7-day-old fly fed on normal food (middle) and 7-day-old starved fly (bottom). Each ovary is observed under dark field (DF; left) and fluorescence illumination (GFP; right). *Yp1::GFP*-derived fluorescence signals are indicated by arrowheads. Fluorescence intensities are measured at three different regions in the ovary; i.e. Area a (inside the white line that delineate the outer limit of the ovary, wherein the yolk is measured); Area b and Area c (regions in germaria located at the anterior tip of the left and right ovarioles, respectively: circled with magenta lines). The circles are of 68.9 μm in diameter (equivalent to 30 pixels) and positioned manually so that it is inscribed at the contact point of the terminal filament and an ovariole that is located at the centre of an ovary. Germaria are occupied by germ stem cells and proliferating daughter cells that are devoid of yolk. Thus, the mean fluorescence intensity measured within germaria (Intensity_b_ + Intensity_c_)/2, represents background noise, which is subtracted from the *Yp1::GFP*-derived fluorescence measured in Area a and Intensity_a_. The subtracted value is normalised for the sizes of measured areas (Size_a_/Size_b_) and, when necessary, also for the body size differences (described by the factor *L*), resulting in Intensity_GFP_. The factor *L* is defined as the body length (the distance between the head tip and the end of the abdominal segment) of a test fly relative to the mean body length of all flies from the relevant control group. Scale bar: 500 μm. **(B)** The proportion of flies in the non-dormancy state (flies carrying at least one yolked oocyte) and dormancy state (flies with unyolked oocytes only) as defined by the conventional method is compared between fed and starved flies. Animals are raised on normal food (see Methods for compositions) at 25°C throughout the larval stage, then divided into three groups at eclosion: the first group is fed normal food (fed flies); the second group is contained in an agar-only medium at 11°C for a week (starved flies); and the third group is sacrificed immediately after eclosion to observe the ovaries. The genotype of flies: *Yp1::GFP/+*. *n* = 20–40. **(C)** The fluorescence intensity values (in a.u.) are compared among the three feeding states. The photoperiod applied was L/D: 12/12 throughout the experiments. The mean ± SEM are indicated. *n* = 20. Statistical significance was evaluated by the Fisher’s exact test **(B)** and the Kruskal–Wallis test with post-hoc test **(C)**: ^**^p < 0.01 and ^****^p < 0.0001.

To accomplish accurate and fast quantification of yolk in the ovary, we used *Yp1::GFP*, a fly-TransgeneOme line that produces full-length Yp1 tagged with superfolder-GFP at its C-terminus, for visualisation of Yp1 (Figures 1A–C; Sarov et al., 2016). This paper focuses on the effects of feeding status and temperature, because these are major factors that affect ovarian maturation, while the photoperiod has only a marginal effect in *D. melanogaster* (Saunders and Bertossa, 2011, but see Nagy et al., 2018). We previously showed that the proportion of female flies in reproductive dormancy as defined by the conventional criterion dramatically increases among starved flies compared to that among fed flies when both fly groups were kept at 11°C for a week after adult emergence (Ojima et al., 2018). We confirmed that this is also the case for *Yp1::GFP* heterozygotes (Figures 2A,B). When the amount of yolk in the ovary was quantified by the GFP fluorescence intensity of the same *Yp1::GFP* flies, starvation significantly reduced yolk accumulation (Figure 2C). Additionally, we found no GFP fluorescence in the ovary of female flies immediately after eclosion (Figures 2A,C). Fluorescence intensity measurements revealed that the amount of yolk accumulated in the ovary varies from fly to fly (Figure 2C), in contrast with the dormancy estimation obtained using the conventional method, which yielded no information about the maturity of ovaries in each ‘non-diapausing’ fly (Figure 2B). We consider that the fluorescence intensity-based measurement of yolk accumulation is superior to the conventional method, because the new method, but not the conventional method, provides data on the variability in oogenesis among fly individuals.

**Figure 3:**
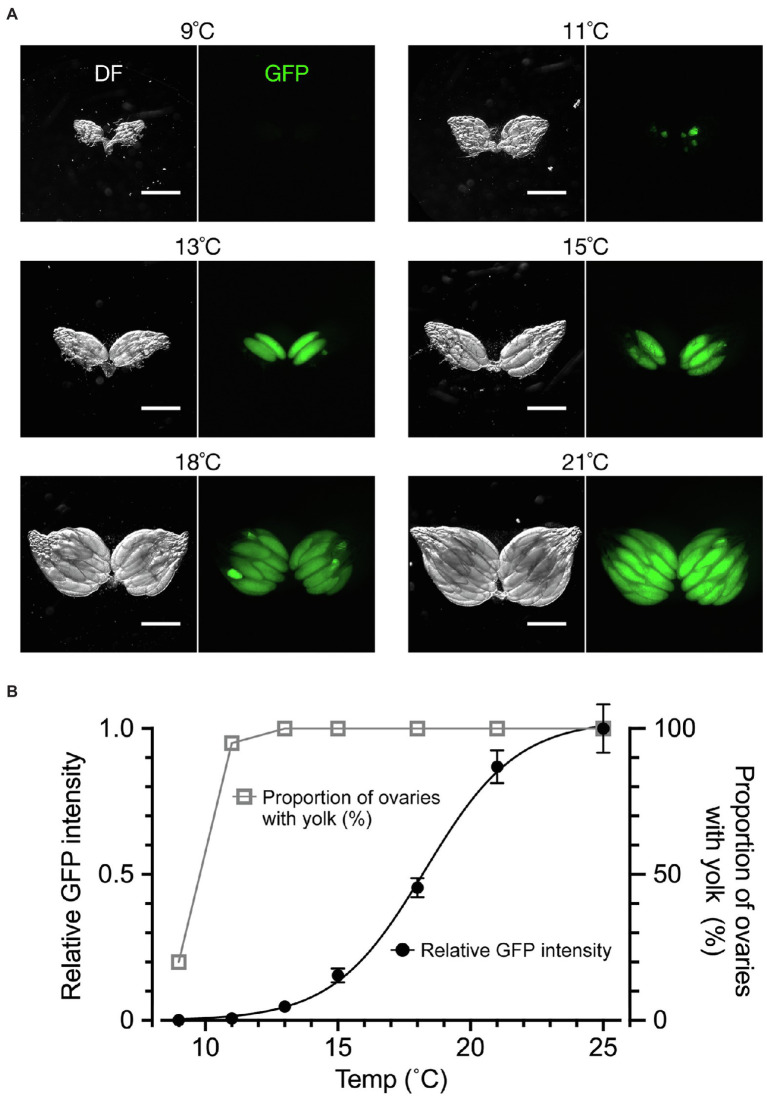
The temperature dependence of yolk accumulation. **(A)** Representative images of ovaries (DF: dark field; GFP: fluorescence) from flies kept for 7 days in vials with normal food at the indicated temperature after emergence. Scale bar: 500 μm. **(B)** Temperature–yolk accumulation relationships. The ordinate scale on the right: the proportion of ovaries with yolk-containing egg chambers (open triangles and grey line) representing the non-dormancy rate according to the conventional method. The ordinate scale on the left: the mean and SEM for fluorescence intensity values (in a. u.; filled circles and black line). *n* = 20. The line represents a four parameter logistic curve in which the maximum value is 1.030, the minimum value is −0.001, IC_50_ is 18.28 and the slope factor is 0.2504. The animals were fed on a normal cornmeal–yeast diet (see Methods for compositions) throughout all stages. They were kept at 25°C during the larval stage and then kept at the indicated temperature after emergence. The photoperiod applied was L/D: 12/12 throughout the experiment. The genotype of flies: *Yp1::GFP/+*.

We next examined the effect of temperature, to which the flies were exposed for a week after adult emergence, on yolk accumulation and dormancy induction (Figure 3). When the dormancy state was judged by the conventional method, ~20% of flies were in the non-dormancy state at 9°C; as the temperature increased, the proportion of flies in the non-dormancy state sharply increased, reaching to 100% at 13°C (Figures 3A,B). Notably, when using the conventional method, even when only a single egg chamber within the entire ovary contained a trace amount of yolk, the fly was classified into the non-dormancy group. In contrast, fluorescence-based GFP measurements of ovarian yolk accumulated in the same flies resulted in a temperature-response curve spanning a much wider temperature range: yolk deposition occurred at over 11°C and reached the maximum at 25°C (Figure 3B). The less steep temperature-response relationship obtained by fluorescence measurements likely reflected a gradient in the maturation levels among egg chambers in the ovary, which may be a useful marker to detect developmental variabilities in the yolk incorporation process across chambers. We conclude that the quantification of yolk accumulation by GFP has advantages over the conventional method, because it monitors the maturation levels of all egg chambers across the entire ovary and is less vulnerable to outliers.

**Figure 4:**
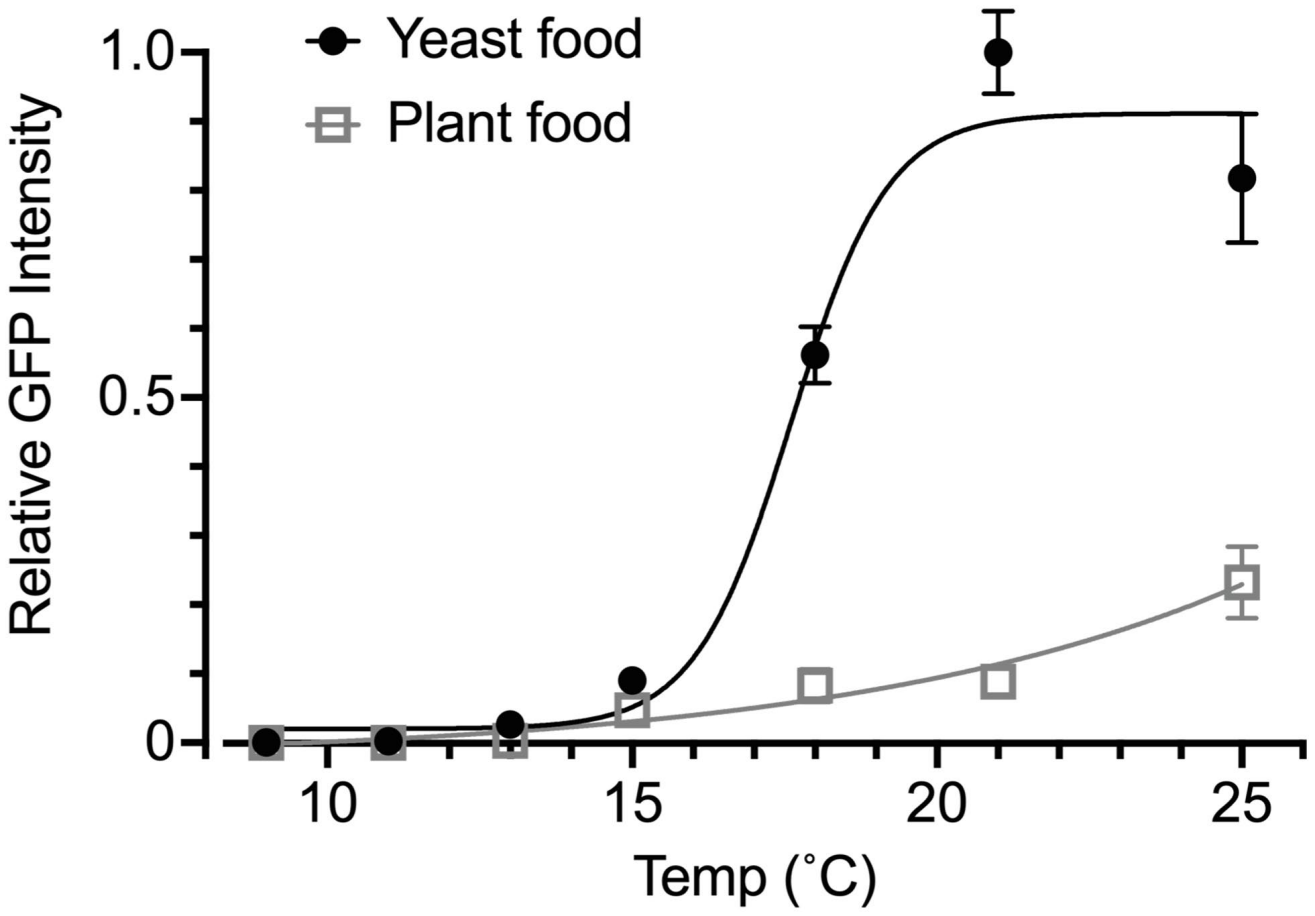
Effects of food quality on temperature-response relationships. The GFP fluorescence intensity was used to measure the yolk content in the ovary of animals fed yeast food or plant food throughout the larval and adult stages. The rearing temperature was 25°C in the larval stage for all tested individuals. The flies were then kept at the indicated temperature at eclosion for 1 week. The curves are four parameter logistic curves with the maximum of 19.64, the minimum of −0.025, IC_50_ of 53.26 and the slope factor of 0.067 for the plant food group (open triangles and grey line) and the maximum of 0.911, the minimum of 0.02, IC_50_ of 17.61 and the slope factor of 0.549 for the yeast food group (filled circles and black line). The animals were kept under the photoperiod of L/D: 12/12 throughout the experiment. The genotype of flies: *Yp1::GFP/+*. The mean ± SEM are shown. *n* = 6–10.

We also used the *Yp1::GFP* reporter to determine the effect of food quality on temperature-dependent yolk accumulation in the ovary. We compared the yolk levels between female flies that were fed on yeast food and those that were fed on plant food. The plant food was shown to confer cold tolerance on flies at the expense of egg production in those flies (Brankatschk et al., 2018). These two diets, which were developed by Brankatschk et al. (2014), had similar proportions of calories derived from protein, lipid and carbohydrate but differed in lipid composition: yeast food contained saturated and mono-unsaturated fatty acids of 14–18 carbon units, whereas plant food contained longer polyunsaturated fatty acids (Brankatschk et al., 2014). In addition, the yeast food contained fungal sterols, whereas the plant food contained phytosterols (Brankatschk et al., 2014). Intriguingly, female flies that consumed plant food exhibited a very low level of yolk accumulation at all temperatures examined (Figure 4). We concluded that the measurement of fluorescence signals derived from *Yp1::GFP* offers a reliable, sensible means for the quantification of yolk accumulated in the ovary, even when the yolk amount is extremely small.

## Discussion

In this study, we demonstrated that the *Yp1::GFP* transgenic line serves as a useful marker of the amount of yolk deposited in the ovary in female *D. melanogaster*. By integrating GFP fluorescence signals derived from *Yp1::GFP* for the entire ovary, the level of ovarian maturation of each fly can be described on an absolute scale. To define reproductive dormancy, the conventional method adopted the criterion that the fly has no egg chamber with yolk (i.e. stage 7 or younger): if there exists one egg chamber with yolk (i.e. stage 8 or older), then the fly is not in the dormancy state, regardless of the conditions of other egg chambers in the ovary (Saunders et al., 1989). We propose redefining the dormancy state based on the evaluation of overall maturation levels of egg chambers in the ovary, as aided by quantitative measurements of the yolk amount in the ovary with *Yp1::GFP*. The temperature-response relationships obtained by GFP fluorescence measurements can be well approximated by sigmoidal curves; therefore, the maximal and half maximal values and the slope factors of the sigmoidal functions may be useful variables for describing the yolk accumulation dynamics of the fly. Diapause is not a simple retardation of growth but rather an active process to enhance stress resistance. It is therefore envisaged that relationships between the temperature and yolk accumulation in diapausing flies would follow dynamics distinct from those in growth retarded flies.

Because the fluorescence-based method proposed here measures signals across the entire ovary, the staging of single egg chambers will not be required for defining the dormancy state. By contrast, in the conventional method, the absence or presence of progression from previtellogenic stage 7 to vitellogenic stage 8 was the key event that distinguishes the dormancy state from the non-dormancy state (Saunders et al., 1989; Kubrak et al., 2014). This traditional view was recently challenged based on the observation that yolk accumulation started before adult eclosion; thus, some egg chambers are already in stage 8 at emergence, which in turn degenerates upon exposure to diapausing conditions (Lirakis et al., 2018). Lirakis et al. (2018) argued that reproductive dormancy is maintained by the suspended uptake of circulating yolk proteins of fat-body origin that otherwise induce a rapid increase in yolk mass, which is characteristic of stage 10. Because of this, flies in the dormancy state may not carry ovaries that harbour stage 10 or older egg chambers. In the present work, *Yp1::GFP* reporter expression was not detected in the ovaries of females immediately after eclosion, which is contrary to reports that egg chambers of stage 8 or older are observed in newly emerged females. It remains to be examined whether Yp2, Yp3 or some other constituents contribute to the yolk that is present in the ovary immediately after eclosion. The *Yp1* and *Yp2* genes align tandemly on chromosome-X in a head-to-head orientation, whereas the *Yp3* gene is located at some distance from the *Yp1* and *Yp2* genes on the same chromosome. These three genes are expressed coordinatively in the fat body and ovaries (Garabedian et al., 1985; Logan and Wensink, 1990; Hutson and Bownes, 2003). It remains to be determined whether the expression of Yp2 and Yp3 responds to food and temperature in a similar way as Yp1 expression.

Although measuring *Yp1::GFP* fluorescence allows the quantitative description of ovarian maturation, there are limitations inherent in the new method. First, unlike in the conventional method, fundamental skills for operating a fluorescent microscope are required. Second, preparing tissue samples for fluorescence measurements is more labour-intensive than the conventional routine of simply dissecting and observing the ovaries. Third, differences in the settings of fluorescent measurements across researchers might complicate the comparisons of data from different research groups. Fourth, the *Yp1::GFP* transgene must be integrated, by genetic crosses, into the genome of all fly strains to be examined: different strains typically have distinct genetic backgrounds, which can be standardised only after laborious outcrossing.

Yolk proteins are not just nutrients for embryos; for example, they participate in the proper formation of an embryonic axis by supporting Oskar functions (Tanaka et al., 2021). The precise quantification of yolk proteins is thus pivotal for a thorough understanding of adaptive mechanistic changes of oogenesis under variable environmental conditions.

## Data Availability Statement

The data sets generated for this paper are available from the corresponding author upon reasonable request.

## Author Contributions

DY and YH: conceptualisation, writing – review and editing, and funding acquisition. YH: methodology, investigation, and visualisation. DY: writing – original draft, supervision, and project administration. All authors contributed to the article and approved the submitted version.

## Funding

This work was supported in part by the Grants-in-Aid for Scientific Research from the Ministry of Education, Culture, Sports, Science and Technology (21H04790 to DY; 20 K15846 to YH), an Act-X grant from Japan Science and Technology Agency (JPMJAX191B), the Academic Research Grant Project from Hyogo Science and Technology Association and TRIAL grant from NICT to YH.

## Conflict of Interest

The authors declare that the research was conducted in the absence of any commercial or financial relationships that could be construed as a potential conflict of interest.

## Publisher’s Note

All claims expressed in this article are solely those of the authors and do not necessarily represent those of their affiliated organizations, or those of the publisher, the editors and the reviewers. Any product that may be evaluated in this article, or claim that may be made by its manufacturer, is not guaranteed or endorsed by the publisher.
